# Supplementary material for: Explainable machine learning for predictive modeling of blowing snow detection and meteorological feature assessment using XGBoost-SHAP
Source: PLoS One. 2025 Mar 28;20(3):e0318835. doi: 10.1371/journal.pone.0318835 (PMC11952239; doi:10.1371/journal.pone.0318835)
Supplement: S1 Fig — (DOCX) [file pone.0318835.s001.docx]

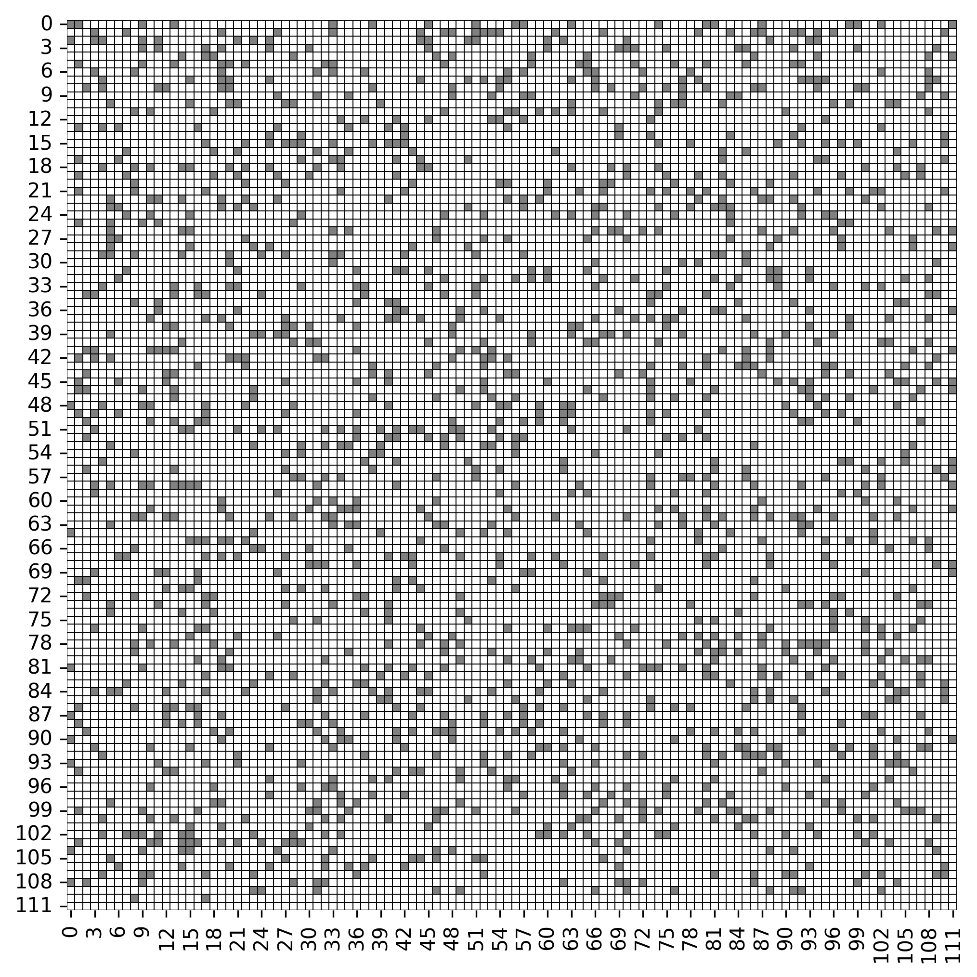


S1 Fig. Observed wind-blown snow events (gray) and non-wind-blown snow events (white) in the validation set (FBER), with each cell representing one hour.
