# Supplementary material for: Explainable machine learning for predictive modeling of blowing snow detection and meteorological feature assessment using XGBoost-SHAP
Source: PLoS One. 2025 Mar 28;20(3):e0318835. doi: 10.1371/journal.pone.0318835 (PMC11952239; doi:10.1371/journal.pone.0318835)
Supplement: S2 Fig — (DOCX) [file pone.0318835.s002.docx]

| 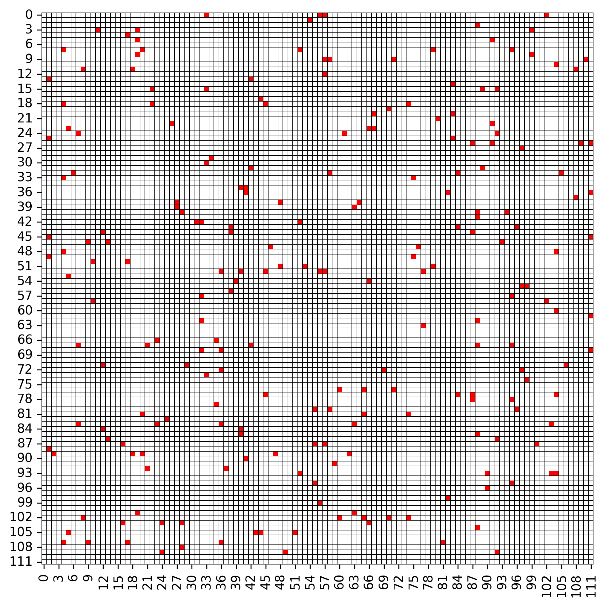  (a) | 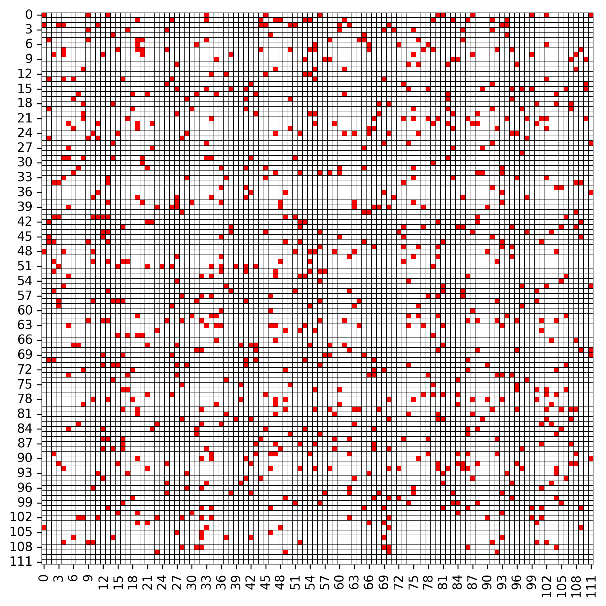  (b) |
| --- | --- |
| 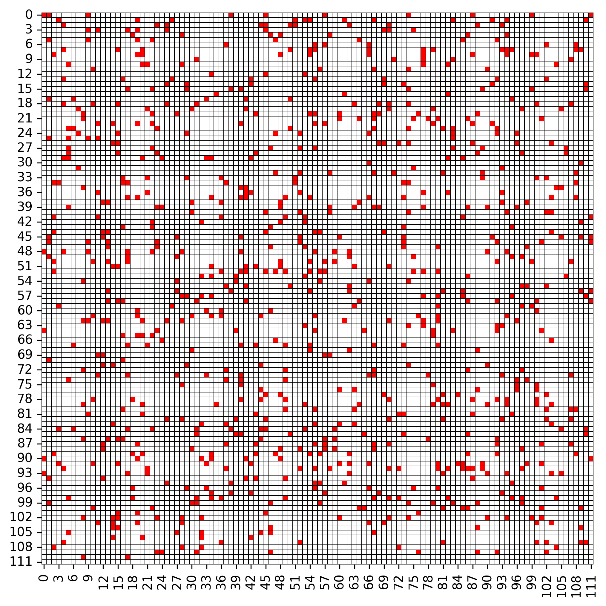  (c) | 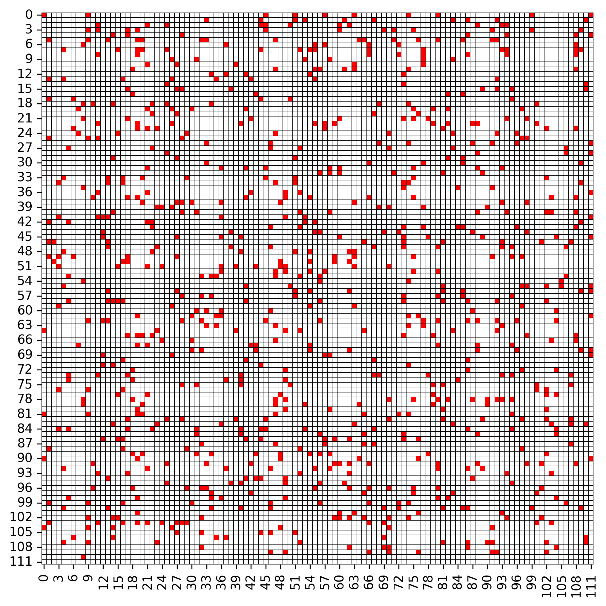  (d) |

S2 Fig. Predicted from (a) XGBoost, (b) SVM, (c) RF, and (d) GBDT models compared against the observed validation set (FBER), with white cells indicating matches and red cells indicating mismatches.
