# Supplementary material for: Explainable machine learning for predictive modeling of blowing snow detection and meteorological feature assessment using XGBoost-SHAP
Source: PLoS One. 2025 Mar 28;20(3):e0318835. doi: 10.1371/journal.pone.0318835 (PMC11952239; doi:10.1371/journal.pone.0318835)
Supplement: S1 Table — (DOCX) [file pone.0318835.s003.docx]

S1 Table. Statistical characteristics of the cleaned training data.

|  |  | **WS-AVG** | **WS-MAX** | **WD-MIN** | **WD-MAX** | **RYS** | **RAS** | **RAI-6** | **RAI-24** | **RI-MIN** | **RI-MAX** | **SD** | **SH** | **AT** | **AH** |
| --- | --- | --- | --- | --- | --- | --- | --- | --- | --- | --- | --- | --- | --- | --- | --- |
| FBER | **Mean** | 7.1 | 13.6 | 127.6 | 184.6 | 1119.8 | 0.5 | 0.2 | 0.9 | 0.0 | 0.0 | 0.1 | 36.5 | -1.3 | 66.7 |
|  | **Min** | 0.4 | 0 | 0 | 0 | 0 | 0 | 0 | 0 | 0 | 0 | 0 | -13.2 | -21 | 0 |
|  | **25%** | 0.8 | 6.2 | 21 | 71 | 230.2 | 0 | 0 | 0 | 0 | 0 | 0 | 1.1 | -5 | 45 |
|  | **50%** | 3.5 | 10.2 | 111 | 186 | 1042.4 | 0 | 0 | 0 | 0 | 0 | 0 | 27.2 | -1 | 70 |
|  | **75%** | 10 | 18.5 | 207 | 304 | 1798.6 | 0.5 | 0 | 0.9 | 0.0 | 0.0 | 0 | 56.6 | 2.6 | 92 |
|  | **Max** | 40 | 40 | 360 | 360 | 4107.8 | 280.6 | 106.8 | 113.2 | 8.2 | 42 | 1 | 245 | 16.9 | 100 |
|  | **Std** | 9.0 | 11.2 | 110.8 | 120.8 | 950.0 | 3.9 | 1.3 | 3.3 | 0.1 | 0.3 | 0.3 | 39.4 | 5.6 | 25.9 |
| FGIE | **Mean** | 11.2 | 12.3 | 167.4 | 195.1 | 223.4 | 0.7 | 0.4 | 1.5 | 0.0 | 0.1 | 0.1 | 70.2 | 0.6 | 77.2 |
|  | **Min** | 0 | 0 | 0 | 0 | 0 | 0 | 0 | 0 | 0 | 0 | 0 | -31.9 | -22.4 | 5.2 |
|  | **25%** | 1.6 | 7 | 98 | 115 | 52.4 | 0 | 0 | 0 | 0 | 0 | 0 | 17.9 | -3.4 | 59.1 |
|  | **50%** | 10.6 | 11.4 | 157 | 223 | 125.4 | 0 | 0 | 0 | 0 | 0 | 0 | 70.2 | 0.6 | 85 |
|  | **75%** | 18.5 | 16.7 | 258 | 283 | 329.8 | 0 | 0.2 | 1.5 | 0 | 0 | 0 | 102.5 | 4.5 | 98.8 |
|  | **Max** | 40 | 40 | 360 | 360 | 998 | 123.2 | 34.4 | 64.4 | 8 | 10.4 | 1 | 310 | 22 | 100 |
|  | **Std** | 9.3 | 8.8 | 99.2 | 102.6 | 234.9 | 4.4 | 1.4 | 3.9 | 0.2 | 0.3 | 0.3 | 56.7 | 5.7 | 23.7 |
| FHUE | **Mean** | 11.2 | 18.0 | 141.3 | 183.1 | 941.1 | 0.5 | 0.3 | 1.1 | 0.0 | 0.1 | 0.1 | 65.5 | 1.1 | 71.1 |
|  | **Min** | 0.4 | 0 | 0 | 0 | 0 | 0 | 0 | 0 | 0 | 0 | 0 | -197.7 | -20.8 | 1.6 |
|  | **25%** | 1.8 | 8.6 | 23 | 70 | 137.8 | 0 | 0 | 0 | 0 | 0 | 0 | 16.4 | -2.3 | 55 |
|  | **50%** | 7.6 | 15.3 | 141.3 | 183.1 | 922 | 0 | 0 | 0 | 0 | 0 | 0 | 64.9 | 1.1 | 76.9 |
|  | **75%** | 17.3 | 25.9 | 214 | 298 | 1082.4 | 0.5 | 0 | 1.1 | 0 | 0.1 | 0 | 99.2 | 4.7 | 92.3 |
|  | **Max** | 40 | 40 | 360 | 360 | 3583.1 | 221 | 106.6 | 153 | 25 | 36.8 | 1 | 260 | 20.6 | 98.2 |
|  | **Std** | 11.0 | 11.3 | 113.9 | 118.3 | 882.8 | 4.2 | 1.8 | 5.0 | 0.2 | 0.5 | 0.3 | 52.6 | 5.2 | 23.7 |
