# Supplementary material for: Explainable machine learning for predictive modeling of blowing snow detection and meteorological feature assessment using XGBoost-SHAP
Source: PLoS One. 2025 Mar 28;20(3):e0318835. doi: 10.1371/journal.pone.0318835 (PMC11952239; doi:10.1371/journal.pone.0318835)
Supplement: S2 Table — (DOCX) [file pone.0318835.s004.docx]

S2 Table. Spearman correlation coefficients between the considered parameters and wind-blown snow events.

|  | **Spearman Correlation** | | |
| --- | --- | --- | --- |
| **Variable** | **FBER1** | **FGIE1** | **FHUE1** |
| WS-AVG | 0.418 | 0.159 | 0.399 |
| WS-MAX | 0.352 | 0.237 | 0.411 |
| AH | 0.247 | 0.329 | 0.270 |
| SH-SEG1 | 0.085 | 0.040 | 0.064 |
| RAI-24 | 0.073 | 0.075 | 0.056 |
| RAI-6 | 0.065 | 0.051 | 0.052 |
| SH-SEG2 | 0.060 | 0.028 | 0.068 |
| RAS | 0.039 | 0.020 | 0.051 |
| RI-MAX | 0.039 | 0.019 | 0.046 |
| RI-MIN | 0.012 | 0.000 | 0.019 |
| WD-MIN | 0.009 | 0.096 | 0.168 |
| RYS | -0.003 | 0.032 | -0.018 |
| WD-MAX | -0.079 | 0.063 | 0.132 |
| AT | -0.137 | -0.230 | -0.202 |
