# Supplementary material for: Explainable machine learning for predictive modeling of blowing snow detection and meteorological feature assessment using XGBoost-SHAP
Source: PLoS One. 2025 Mar 28;20(3):e0318835. doi: 10.1371/journal.pone.0318835 (PMC11952239; doi:10.1371/journal.pone.0318835)
Supplement: Attachment — Submitted filename: 01_Response to Reviewers.docx [file pone.0318835.s009.docx]

| Journal | PLOS ONE |
| --- | --- |
| Manuscript title (new) | Explainable Machine Learning for Predictive Modeling of Blowing Snow Detection and Meteorological Feature Assessment Using XGBoost-SHAP |
| Manuscript ID | PONE-D-24-48529 |
| First submission date | Oct 26 2024 |
| Corresponding author | *Feng Wang* (21114126@bjtu.edu.cn)  School of Civil Engineering, Beijing Jiaotong University, Beijing, China |

Dear Reviewers and Academic Editor,

We would like to express our sincere gratitude to the reviewers and the academic editor for their valuable feedback and constructive suggestions. We have carefully reviewed each of the comments and have provided detailed responses and revisions accordingly. Your insightful recommendations have significantly enhanced the quality of our manuscript, particularly in areas such as figure formatting, selection of training parameters, presentation of training data, model evaluation, and the discussion of results, among others.

In this document, we provide a thorough response to your comments, following the order of

- Reviewer #1 (Page 2),
- Reviewer #2 (Page 18),
- and the Academic Editor (Page 32),

along with the corresponding revisions made to the manuscript. We hope that the revised manuscript now comprehensively addresses your concerns and meets the standards set by *PLOS ONE*.

Sincerely,

Feng Wang

# Reviewer #1

[Comment #1] The innovation of the work should be explained in a better way in the introduction section

[Response #1] Thank you for your valuable feedback. Based on your suggestion, we have further clarified and articulated the innovation of the study. Indeed, existing research on blowing snow prediction has some limitations, particularly in terms of applying data-driven models and comprehensively analyzing meteorological features. Therefore, the innovation of this study lies in proposing a more detailed and accurate predictive modeling approach for blowing snow events using the XGBoost-SHAP framework. Unlike traditional methods, we extensively explore various meteorological features within the data and utilize SHAP analysis to reveal the impact and interactions of these features on blowing snow events, thereby enhancing both the interpretability and accuracy of the model.

To better highlight the novelty of our work, we have revised Section 1.3 (Contributions) to more precisely outline the unique contributions of this study.

(The dividing line indicates the beginning or end of the content referenced from the revised manuscript, with all modified content specially highlighted in red.)

-------------------------

(line 123 of the Revised Manuscript with Track Changes)

Despite progress in research on forecasting snow drift events, several challenges remain in establishing the relationship with meteorological conditions. ~~Existing studies often rely on traditional methods, resulting in significant discrepancies in threshold wind speeds across different studies, as well as a lack of standardized methods for determining threshold wind speeds. These issues further limit the applicability of the research and make it difficult to generalize findings across various regions and climatic conditions.~~ One major issue is that existing studies often rely on traditional empirical models that oversimplify the complex interactions between meteorological variables, leading to significant discrepancies in threshold wind speed estimates across different regions and climatic conditions. These traditional approaches typically treat wind speed and other environmental factors as isolated variables, overlooking their dynamic and multifaceted interactions. As a result, threshold wind speed values, which are critical for predicting snow transport and drift initiation, often vary widely between studies.

Moreover, existing research generally lacks a standardized method for defining and measuring threshold wind speeds. Field-based studies have reported widely differing values for the wind speed required to initiate snow transport, with discrepancies often attributed to differences in experimental setup, measurement techniques, and regional environmental conditions. This inconsistency not only complicates the validation of snow drift models across different regions but also limits their applicability to broader climatic contexts. As a result, current models often fail to generalize effectively, making it difficult to predict snow drift behavior reliably in diverse environments.

These challenges underscore the need for a more nuanced, data-driven approach that can better account for the complex, multifactorial nature of snow drift dynamics. To tackle these issues, the main objective of this study is to assess the performance of XGBoost in detecting the occurrence of blowing snow and to analyze the importance of individual features for detection using SHAP. …

-------------------------

[Comment #2] Consider a better title for this study.

[Response #2] Thank you for your insightful suggestion. We acknowledge that the initial title of our manuscript lacked sufficient specificity. In response, we have revised the title to provide a clearer and more precise description of the focus and methodology of the study.

The original title, "Explainable Machine Learning Model for Blowing Snow Detection and Feature Analysis Using XGBoost-SHAP," was broad and did not fully capture the scope of the work, particularly the emphasis on predictive modeling and the in-depth analysis of meteorological features. To address this, we have updated the title to:

Explainable Machine Learning for Predictive Modeling of Blowing Snow Detection and Meteorological Feature Assessment Using XGBoost-SHAP

[Comment #3] Why is the XGBoost method used? Isn't it better to use other models machine learning and compare the results? For this purpose, you can get help from the following article.

https://doi.org/10.1007/s11356-024-32228-x

[Response #3] As you pointed out, it is indeed essential to compare the results of different machine learning models and explain why we have chosen XGBoost for this study, along with its advantages. Similar to the approach adopted by Niknam et al. (2024), who compared the performance of time series models such as ARIMA, Simple_RNN, LSTM, CNN, GRU, and MLP for ongoing monitoring and prediction of water quality, we have also conducted a model comparison in our study. Specifically, in section 4.1 "Model Evaluation", we compare the performance of XGBoost with other classical classification models, such as SVM, RF, and GBDT, on the same dataset. The performance metrics used for this comparison include the confusion matrix, Precision, Recall, and F1-score.

Our results show that while XGBoost and the other three models (SVM, RF, and GBDT) exhibit similar performance in identifying non-snowdrift events, XGBoost outperforms the other models in detecting snowdrift events, especially when the sample size is smaller. This is primarily because XGBoost utilizes a Gradient Boosted Decision Tree (GBDT) ensemble learning framework, which enhances predictive accuracy by combining multiple weak learners (i.e., decision trees). Compared to SVM, RF, and GBDT, XGBoost incorporates additional regularization terms (such as L1 and L2 regularization) during training, helping to prevent overfitting and optimize the learning rate and tree structure. These features allow XGBoost to better capture complex patterns and subtle feature differences in smaller datasets, such as the snowdrift events in our study.

Furthermore, XGBoost’s iterative correction mechanism, where each tree not only addresses the errors of the current dataset but also corrects the mistakes made by the previous trees, is particularly useful for handling class imbalance. In our classification task, snowdrift events are relatively rare, and XGBoost’s ability to adjust the model iteratively improves its recognition of minority classes, especially when sample sizes are small. This gives XGBoost a significant edge in our research, where detecting snowdrift events is crucial.

In contrast, SVM’s performance may be limited by the choice of kernel function and its sensitivity to class imbalance. While adjusting the C value and kernel function can improve classification performance, SVM is generally more sensitive to noise and class imbalance. Similarly, although RF and GBDT offer stable predictions in many cases, they may not be as efficient as XGBoost in correcting errors during model training, particularly in handling rare classes like snowdrift events. This limitation can lead to a decrease in performance when recognizing these less frequent events.

In conclusion, the strengths of XGBoost lie in its powerful ensemble learning framework, regularization mechanisms, and its ability to adaptively adjust when faced with class imbalance and small sample sizes. These factors make XGBoost particularly well-suited for classifying snowdrift events in our study, where high accuracy is essential, especially when the sample size is limited.

To strengthen the quality of the manuscript, we have incorporated these points into the revised version of section 4.1 "Model Evaluation."

-------------------------

(line 436)

Due to the class imbalance in wind-blown snow events (Class 1), the models face greater difficulty in predicting these events, especially with significant discrepancies between Precision and Recall. Among the models, XGBoost demonstrates the most stable performance when handling Class 1. Across the three resampled datasets (FBER, FGIE, FHUE), Precision ranges from approximately 0.77 to 0.80, Recall from 0.84 to 0.87, and the F1-score is around 0.80 to 0.83.

The superior performance of XGBoost can be attributed to several key factors, especially when compared to models such as SVM, RF, and GBDT. XGBoost utilizes an ensemble learning framework, which aggregates multiple weak learners (decision trees) to enhance predictive accuracy. Furthermore, XGBoost incorporates an iterative correction process in which each tree not only focuses on correcting the errors from the current dataset but also refines the mistakes made by previous trees. This mechanism proves particularly effective for handling imbalanced datasets, such as in the case of wind-blown snow events, where the target class (Class 1) is underrepresented. By progressively refining the model’s predictions, XGBoost is better equipped to capture complex patterns and subtle feature differences, especially in smaller sample sizes. This iterative adjustment enables the model to handle the challenges posed by class imbalance and ensures that it can identify minority class events with greater accuracy.

-------------------------

[Comment #4] The quality of Figure 1 should be improved. Also, the figure should have latitude and longitude.

[Response #4] Thank you for your helpful suggestion. We appreciate that both you and the academic editor raised the same concern regarding the quality of Figure 1. In response, we have improved the figure in the revised manuscript and adding the necessary latitude and longitude coordinates.

Fig 1. Study area and locations of selected meteorological stations

[Comment #5] Other variables can also affect the blowing of snow. You can consider different variables and then identify more effective parameters with sensitivity analysis using different methods such as Monte Carlo and use them in the machine learning method. You can get ideas from the following articles

https://doi.org/10.1016/j.coldregions.2022.103682

https://doi.org/10.1007/s11600-023-01177-3

[Response #5] We agree that there are numerous variables that could influence the initiation of snow drift, such as the angle of repose, snow particle size distribution, snow density, and thermal permittivity (Goodarzi et al., 2024). However, this study focused on a more limited set of parameters, namely wind speed, temperature, humidity, precipitation, and snow depth. We would like to provide further clarification on our choice of parameters for this study:

- Interdependencies of Variables: Many of the variables considered in snow drift prediction are interrelated. For instance, higher temperatures tend to reduce the number of smaller snow particles, while higher humidity can increase the angle of repose, and temperature fluctuations can affect thermal permittivity. While incorporating additional parameters could enhance the model, focusing on the most significant ones is critical for maintaining the model's interpretability and performance. This approach is aligned with the feature analysis presented in Section 4.2, where we rank the importance of different variables in predicting blowing snow events.
- Practicality and Applicability: The objective of this study is to develop a predictive model for snow drift that can be implemented in the field. Including too many parameters, especially those that require specialized instruments or complex measurement techniques, could reduce the practicality and applicability of the model. Therefore, we concentrated on easily measurable, commonly available parameters that can be directly observed in operational settings, as opposed to more complex variables that might require specialized equipment.

Taking all these considerations into account, we believe that the chosen parameters are sufficient to develop a robust and practical predictive model for blowing snow events. To further clarify this point, we have added additional explanations in Section 2.1 of the revised manuscript, where we explain why we focused on these basic variables, such as temperature, humidity, wind speed, and precipitation, as included in the ISAW dataset.

-------------------------

(line 172)

Meteorological data and snow flux were recorded at each station with an hourly resolution. The specific variable names and their descriptions are listed in Table 1. This study focused on a more selective set of parameters, including wind speed, temperature, humidity, precipitation, etc. While several additional variables, such as the angle of repose, snow particle size distribution, snow density, and thermal permittivity, have been identified in the literature as influencing snow characteristics (Goodarzi et al., 2024), they were not considered in the present study. This choice was primarily driven by the practical considerations of real-time field application, as many of these additional variables require specialized instruments or complex measurement techniques, which could limit the model’s operational applicability. Moreover, many of the variables relevant to snow drift prediction are interrelated, which introduces further complexity in their integration and interpretation within a predictive framework.

In Table 1, wind speed and direction were measured at a height of 3.5 meters using the Wind Monitor (YOUNG Model 05103). Precipitation intensity was recorded using a Campbell Scientific ARG100 tipping-bucket rain gauge, while snow depth was measured with an SR50 ultrasonic sensor. Snow depth measurements were taken from two different areas near the meteorological stations to account for the spatial variability of snow accumulation and to improve the representativeness of the results, which can be less reliable when measured at a single point.

Goodarzi M R, Sabaghzadeh M, Mokhtari M H. Impacts of aspect on snow characteristics using remote sensing from 2000 to 2020 in Ajichai-Iran[J]. Cold Regions Science and Technology, 2022, 204: 103682.

-------------------------

[Comment #6] The quality of Figure 5 and 6 is not good and the numbers are not visible.

[Response #6] Thank you for your feedback. Both you and Reviewer #2 raised concerns regarding the clarity of Figures 5 and 6. We believe the issue arises from the lower resolution of the images in the initial submission, which were automatically generated by the Editorial Manager^®^ system in the *PONE-D-24-48529.pdf* file. These images are preview versions with reduced clarity. However, if the figures are accessed directly by clicking the "*Click here to access/download; Figure; Fig 5.png*" link in the corresponding page, the original high-resolution source files of Figures 5 and 6 can be viewed, which exhibit excellent clarity.

Therefore, we anticipate that the issue of numbers being unclear in Figures 5 and 6 will not persist in the later stages of the review process. We have attached the high-resolution versions of Figures 5 and 6 below for reference in this resubmission.

If this does not address the concerns you have, we are more than willing to further revise Figures 5 and 6 based on any specific suggestions you may have.

Fig 5. SHAP Summary Plot：(a) FBER; (b) FGIE; (c) FHUE

Fig 6. SHAP force plot showing feature impacts for individual samples: (a), (b), (c) no-blown snow event, and (d) wind-blown snow event

[Comment #7] Calculate the statistical characteristics of the data used and show that in a table.

[Response #7] Thank you for your valuable suggestion. We agree that providing the statistical characteristics of the training data is essential for offering readers a clearer understanding of the dataset's overall properties, as is common practice in many studies. However, due to the substantial size of the statistical characteristics table, we have included this information as *Supplementary Information* in the revised manuscript. Specifically, the details are provided in *Supporting Information*, S1 Table, titled "Statistical characteristics of the cleaned training data."

-------------------------

(line 254)

Previous studies have shown that the FlowCapt sensor is sensitive to moving soil particles, which can lead to false detections of wind-blown snow events, introducing significant uncertainty into the monitoring data[5]. By analyzing snow depth data from different years at various stations, it was found that snow depth remained nearly zero between May and October each year. Therefore, only data from the winter and spring seasons (November to April) were used in this study, minimizing the uncertainty caused by false detections related to soil particles.

Detailed supplementary information is essential to enhance transparency and provide a thorough understanding of the dataset, as well as its relevance to the development of the wind-blown snow prediction model. Specifically, Supporting Information S1 Table summarizes the statistical characteristics of the cleaned data, prepared in accordance with the defined processing criteria.

-------------------------

[Comment #8] Section 3.1 requires more references in the text

<https://doi.org/10.1111/wej.12939>

[Response #8] Thank you for your valuable suggestion. Upon carefully reviewing Section 3.1, we identified a gap in key references, particularly in lines 306-308 of the initial submission, which lacked appropriate supporting citations. As you correctly pointed out, the study by Goodarzi et al. (2024) compares XGBoost with other models such as ANFIS, RF, MARS, M5P, SVR, and GEP, highlighting that "*the XGBoost method is very consistent with the observational data in the training phase*", which aligns directly with the content presented in line 308 of the manuscript.

| Goodarzi et al. (2024),  "*the XGBoost method is very consistent with the observational data in the training phase*" |  |
| --- | --- |

To address this, we have added the relevant citation to line 308, thereby enhancing the logical coherence of Section 3.1 and ensuring proper academic support for the claims made.

-------------------------

(line 340)

With these improvements, the XGBoost algorithm can efficiently handle ISAW's large-scale field monitoring data, which includes multiple complex and interrelated meteorological features (Goodarzi et al., 2024).

Goodarzi M R, Sabaghzadeh M, Barzkar A, et al. A comparison of machine learning methods for estimation of snow density using satellite images[J]. Water and Environment Journal, 2024, 38(3): 437-449.

-------------------------

[Comment #9] Using the correlation coefficient such as Pearson's correlation coefficient, show the correlation between the considered parameters with snow blowing to create a better view of the data.

[Response #9] Thank you for your valuable suggestion. Including the correlation coefficients is indeed important to preliminarily demonstrate the relationships between the considered parameters and wind-blown snow events, thereby providing readers with a more comprehensive understanding of the training data.

To our knowledge, the dependent variable in this study, representing wind-blown snow events, is a binary variable with values of 0 or 1, indicating whether such events occurred. The independent variables are meteorological parameters (e.g., wind speed, temperature, humidity), which are typically continuous. Given this context, the use of traditional Pearson's correlation coefficient is not well-suited due to its assumptions about linear relationships and the nature of the variables.

Instead, Spearman's rank correlation coefficient was employed, as it is well-suited for handling binary classification data. Spearman’s rank correlation is a non-parametric measure that does not require the data to follow a normal distribution. It effectively captures the relationships between continuous variables and discrete binary variables.

Accordingly, the correlations between the considered parameters and wind-blown snow events, as determined using Spearman’s rank correlation coefficient, are presented in Table S2 of the revised manuscript.

-------------------------

(line 254)

Previous studies have shown that the FlowCapt sensor is sensitive to moving soil particles, which can lead to false detections of wind-blown snow events, introducing significant uncertainty into the monitoring data[5]. By analyzing snow depth data from different years at various stations, it was found that snow depth remained nearly zero between May and October each year. Therefore, only data from the winter and spring seasons (November to April) were used in this study, minimizing the uncertainty caused by false detections related to soil particles.

Detailed supplementary information is essential to enhance transparency and provide a thorough understanding of the dataset, as well as its relevance to the development of the wind-blown snow prediction model. Specifically, Supporting Information Table S1 summarizes the statistical characteristics of the cleaned data, prepared in accordance with the defined processing criteria. Furthermore, S2 Table presents the Spearman correlation coefficients between the analyzed parameters and the outcomes of snow-blowing events, providing insights into the relationships influencing the observed phenomena.

-------------------------

[Comment #10] It is better to use other evaluation criteria to evaluate the accuracy of the model. For this purpose, you can get help from the following reference

https://doi.org/10.3390/w15091650

[Response #10] We have carefully reviewed the reference you provided (Goodarzi et al., 2023). This study investigated the impact of snowmelt on flooding, where the Snow-Covered Area (SCA) from 2013 to 2018 was estimated using daily MODIS images with the assistance of Google Earth Engine. Runoff in the area was then simulated using the Snowmelt Runoff Model (SRM). The accuracy of their model was comprehensively evaluated using multiple statistical indicators, including R^2, Dv, NSE, Ep, and Etp.

The evaluation criteria adopted in Goodarzi et al. (2023) primarily focus on assessing the numerical fit between simulated and observed values, which are commonly used in regression problems for predicting continuous variables. In contrast, our study addresses a typical classification problem, where the primary goal is to evaluate the model's ability to distinguish between positive and negative instances. For this purpose, we utilized Precision, Recall, and F1 Score, which are widely recognized metrics for assessing the predictive performance of binary classification models. This approach aligns with similar studies using classification models, such as Xie et al. (2021) (doi: 10.5194/hess-25-3783-2021), Alsumaidaee et al. (2023) (doi: 10.3390/s23063108), and Parsa et al. (2019) (doi: doi.org/10.1016/j.aap.2019.105405), etc.

To our knowledge, the evaluation criteria currently employed in this study are well-suited for binary predictive models related to drifting snow events. Therefore, we believe that introducing additional evaluation metrics is not immediately necessary.

We hope our response addresses your concerns regarding the evaluation criteria. Meanwhile, we are open to further suggestions and would be pleased to revise the manuscript as needed based on your valuable feedback.

[Comment #11] Add a plot of how the observed data matches the modeled data.

[Response #11] Thank you for your valuable suggestion. In our original manuscript, Table 4 provided an overview of the relationship between the modeled data and the observed data through metrics such as Precision, Recall, and F1-score. However, following your comment regarding “how the observed data matches the modeled data”, we recognize that Table 4 alone does not sufficiently illustrate the differences in model performance. To address this concern, we have added additional visualizations to the Supporting Information section in the revised manuscript.

-------------------------

(line 436)

Due to the class imbalance in wind-blown snow events (Class 1), the models face greater difficulty in predicting these events, especially with significant discrepancies between Precision and Recall. Among the models, XGBoost demonstrates the most stable performance when handling Class 1. Across the three resampled datasets (FBER, FGIE, FHUE), Precision ranges from approximately 0.77 to 0.80, Recall from 0.84 to 0.87, and the F1-score is around 0.80 to 0.83.

To provide a more intuitive comparison of the performance of different models on the same dataset, supplementary figures (S1 and S2 Figs) have been included in the Supporting Information section. S1 Fig. presents the wind-blown snow events (gray) and non-wind-blown snow events (white) within the validation set, which accounts for 20% of the total dataset (FBER). Each unit cell in the figure represents one hour. S2 Fig. compares the predicted results from XGBoost, SVM, RF, and GBDT models against the observed validation set. In this figure, white cells represent instances where the predicted data matches the observed data, while red cells indicate mismatches. The results clearly show that XGBoost demonstrates superior predictive performance compared to the other models, as evidenced by its higher agreement with the observed data.

S1 Fig. Observed wind-blown snow events (gray) and non-wind-blown snow events (white) in the validation set (FBER), with each cell representing one hour.

|   (a) |   (b) |
| --- | --- |
|   (c) |   (d) |

S2 Fig. Predicted from (a) XGBoost, (b) SVM, (c) RF, and (d) GBDT models compared against the observed validation set (FBER), with white cells indicating matches and red cells indicating mismatches.

-------------------------

[Comment #12] Can the results of this study be used for the future and predict future events, such as what was done in the following study? Are these results still correct despite changes such as climate change in the future? It is better to discuss this issue in the discussion section.

https://doi.org/10.1002/joc.8130

[Response #12] Thank you for raising this important point. The applicability of the study’s results to future scenarios, including the prediction of wind-blown snow events, is a critical consideration. The proposed prediction model and findings are based on historical meteorological data and current environmental conditions, which form a solid foundation for forecasting similar events under comparable circumstances.

However, it is acknowledged that external factors such as climate change could introduce significant variations in key parameters, including temperature, precipitation patterns, and wind dynamics, potentially influencing the occurrence and characteristics of wind-blown snow events. While the model captures relationships within the range of observed data, its accuracy and reliability for future predictions may diminish if the underlying climatic conditions deviate substantially from those in the training dataset.

To address this limitation, it is recommended that future applications of the model incorporate updated datasets that reflect ongoing climatic trends. Regular recalibration and validation of the model against current observations will help ensure its continued relevance and accuracy. Additionally, integrating predictive climate models into the framework could enhance its robustness under scenarios of environmental change.

These considerations have been included in the discussion section of the revised manuscript to provide a more comprehensive evaluation of the model’s potential future applications and limitations.

-------------------------

(line 753)

This study has achieved efficient prediction of snow drift events and exploration of feature impacts through machine learning methods, providing new insights and tools for research on snow drift disasters. However, limitations exist, primarily due to data constraints; for instance, features such as topography and snow layer temperature were not included in the analysis, which may affect the comprehensiveness of the results. ~~Future research directions could focus on data quality control to ensure the acquisition of more accurate meteorological data, enhancement of model accuracy by further optimizing existing models to accommodate more complex environmental conditions, and data enrichment by collecting additional variables and data under various meteorological conditions to improve the model's generalization capabilities. These measures will contribute to a deeper understanding and enhanced predictive capability for snow drift events.~~

Moreover, while the proposed prediction model demonstrates strong predictive capabilities within the range of historical meteorological data, its applicability to future scenarios, particularly under the influence of climate change, warrants further discussion. Climate change may lead to substantial shifts in key factors such as temperature, precipitation patterns, and wind dynamics, which could alter the occurrence and characteristics of snow drift events. These changes may reduce the reliability of the model if it is applied without adaptation to evolving environmental conditions. To ensure its continued applicability, periodic updates using newly collected datasets that reflect ongoing climatic trends are essential. Additionally, validating the model with contemporary observations and incorporating predictive climate models could enhance its robustness and adaptability to future scenarios.

Future research should prioritize data quality control to improve the precision of meteorological measurements, as well as expand the scope of analysis by including additional variables such as topography and snow layer temperature. Enhancing model accuracy through the optimization of algorithms to account for more complex environmental interactions is another critical direction. Finally, data enrichment by incorporating diverse datasets from various meteorological conditions will improve the model’s generalization capabilities. These efforts will ensure that the model remains relevant and reliable, providing an improved predictive capability for snow drift events.

-------------------------

# Reviewer #2

[Comment #1] Line 147–154: Given the long-term availability of ISAW data, do the authors plan to make the cleaned and processed dataset publicly accessible for reproducibility and further research?

[Response #1] Thank you for your thoughtful feedback. In Section 2 of the manuscript, the exact source for obtaining ISAW data has been provided (http://iav-portal.com/index.php?nav=iodmisawlist). Additionally, detailed descriptions of the database contents, including the variables, measurement methods and instruments, site selection and spatial distribution, as well as the temporal resolution of the data, have been included. Furthermore, the rules for data cleaning and the statistical characteristics of the cleaned dataset have been thoroughly documented.

Even though, as mentioned above, the cleaned data can be easily obtained, we have still provided the cleaned data in S Files 1, 2, and 3 to facilitate research reproduction or further study by other scholars:

- S1 File: Cleaned FBER site dataset
- S2 File: Cleaned FGIE site dataset
- S3 File: Cleaned FHUE site dataset

-------------------------

(line 781 of the Revised Manuscript with Track Changes)

Acknowledgments

We would like to extend our sincere gratitude to the scientists and engineers involved in ensuring the continuous operation of measurements from the ISAW stations. The observational data collected from these stations can be accessed through the ISAW online database at <http://iav-portal.com/index.php?nav=iodmisawlist>. Additionally, the cleaned dataset in S Files 1, 2, and 3 and all author-generated code in S4 File are provided in the Supporting Information to support reproducibility and further research.

Supporting information

S1 Fig. Observed wind-blown snow events (gray) and non-wind-blown snow events (white) in the validation set (FBER), with each cell representing one hour.

S2 Fig. Predicted from (a) XGBoost, (b) SVM, (c) RF, and (d) GBDT models compared against the observed validation set (FBER), with white cells indicating matches and red cells indicating mismatches.

S1 Table. Statistical characteristics of the cleaned training data.

S2 Table. Spearman correlation coefficients between the considered parameters and wind-blown snow events.

S1 File. Cleaned FBER site dataset

S2 File. Cleaned FGIE site dataset

S3 File. Cleaned FHUE site dataset

S4 File. XGBoost-SHAP model implementation for blown snow detection.

-------------------------

[Comment #2] Line no. 224: How was the threshold of 0.1 g/m²·s for defining blowing snow events determined? Was it empirically derived or based on prior literature?

[Response #2] The threshold value of 0.1 g/m^2·s for defining blowing snow events in this study was determined with reference to widely accepted practices in similar research.

For example, Xie et al. (2021) stated, "In this study, periods of blowing snow occurrence were identified when positive blowing snow flux was observed." The value of 0.1 g/m^2·s corresponds to the minimum positive value detectable by the FlowCapt sensor. He and Ohara (2017) also noted, "The threshold snow flux was set as 0.1 g/m^2·s in this study, and the corresponding wind speed is the threshold wind speed for model validation." These studies provided a solid foundation for adopting the threshold value used in this study.

Thank you for the helpful reminder. The necessary references have been added to Line 244 to ensure clarity and proper citation.

-------------------------

(line 250)

In this study, samples with a snow flux greater than 0.1 g/m2·s were defined as wind-blown snow events (Xie et al., 2021; He and Ohara, 2017),with a wind-blown snow index assigned a value of 1.

Xie Z, Ma W, Ma Y, et al. Decision tree-based detection of blowing snow events in the European Alps[J]. Hydrology and Earth System Sciences, 2021, 25(7): 3783-3804.

He S, Ohara N. A new formula for estimating the threshold wind speed for snow movement[J]. Journal of Advances in Modeling Earth Systems, 2017, 9(7): 2514-2525.

-------------------------

[Comment #3] Line 237–240: Why was SMOTE chosen over other imbalance handling techniques such as ADASYN or Tomek Links? Were comparative tests performed?

[Response #3] Thank you for your insightful question. In our initial selection of techniques for addressing data imbalance, we conducted a preliminary comparison between undersampling methods, represented by Tomek Links, and oversampling methods, represented by SMOTE. The results indicated that SMOTE, as a classic oversampling technique, improved the predictive performance for sparse events, particularly in terms of Recall, which was our primary evaluation metric. However, we did not include a comparison with other oversampling techniques beyond SMOTE in the original analysis.

We agree that including results from other imbalance handling techniques would enhance the representativeness of the study. As a supplement, we incorporated the performance of models trained on datasets processed using ADASYN (an advanced oversampling method) and Tomek Links (an undersampling method). These results are now presented in Table 4 as datasets FBER-a and FBER-t, respectively.

The findings reveal that ADASYN slightly outperformed SMOTE in terms of Recall, while Tomek Links resulted in marginally higher Precision. Overall, the average performance (measured by F1-score) was comparable across the three methods. Importantly, all three techniques led to an approximately 15% improvement in F1-score compared to the original dataset (FBER-o) without any imbalance handling.

Future research could focus on developing new imbalance handling techniques or fine-tuning existing methods through hybrid approaches to further enhance model performance. For this study, we prioritized methods based on the specific characteristics of the dataset. Wind-blown snow events constituted a relatively small portion of the dataset, causing the model to inherently favor classifying non-events (0) more effectively than events (1). By applying a well-established oversampling technique like SMOTE, we sufficiently augmented the minority class (1), enabling the model to better capture the patterns associated with wind-blown snow events.

-------------------------

(line 418)

FBER-o refers to the original dataset, which was not subjected to resampling prior to training, while the other datasets were resampled using SMOTE. FBER-a and FBER-t represent datasets processed with ADASYN and Tomek Links, respectively. Both ADASYN and SMOTE fall under the category of oversampling techniques, with ADASYN being a more advanced approach. In contrast, Tomek Links is an undersampling method. All three are widely used techniques for addressing imbalanced datasets (Swana et al., 2022). Unless otherwise specified, datasets are assumed to have been processed using SMOTE. Furthermore, data from the FBER station was utilized to compare the performance of the model trained with SMOTE sampling against other machine learning models, such as SVM, RF, and GBDT, on this dataset.

Table 4. Performance of the models on field monitoring data

|  |  | Class 0 | | | Class 1 | | |
| --- | --- | --- | --- | --- | --- | --- | --- |
|  | Dataset | Precision | Recall | F1-score | Precision | Recall | F1-score |
| XGBoost | FBER | 0.97 | 0.95 | 0.96 | 0.79 | 0.86 | 0.82 |
|  | FGIE | 0.94 | 0.96 | 0.95 | 0.77 | 0.84 | 0.80 |
|  | FHUE | 0.98 | 0.96 | 0.97 | 0.80 | 0.87 | 0.83 |
|  | FBER-a | 0.98 | 0.96 | 0.97 | 0.77 | 0.88 | 0.82 |
|  | FBER-t | 0.97 | 0.98 | 0.98 | 0.85 | 0.82 | 0.83 |
|  | FBER-o | 0.95 | 0.98 | 0.96 | 0.83 | 0.63 | 0.72 |
| SVM | FBER | 0.95 | 0.99 | 0.97 | 0.77 | 0.45 | 0.57 |
| RF | FBER | 0.95 | 0.98 | 0.96 | 0.68 | 0.79 | 0.73 |
| GBDT | FBER | 0.98 | 0.97 | 0.97 | 0.71 | 0.81 | 0.76 |

~~*FBER-o refers to the original dataset, which has not undergone resampling prior to training, while the other datasets have been resampled (SMOTE).~~

-------------------------

(line 464)

~~Additionally, the~~ The performance of XGBoost on the original FBER-o dataset for Class 1 predictions differs significantly from that on the resampled datasets. After resampling, although the Precision decreased slightly from 0.83 to 0.79–0.80, the Recall improved markedly, rising from 0.63 to 0.84–0.87. This demonstrates that resampling techniques enable the model to better capture the minority class of wind-blown snow events, substantially reducing the number of False Negatives (missed events). This aligns with the critical nature of wind-blown snow hazard prediction, where these events, though infrequent, can cause significant damage. Missing such an event (False Negative) typically carries far graver consequences than issuing a false alarm (False Positive)

Furthermore, a comparison of different resampling methods revealed that ADASYN slightly outperformed SMOTE in terms of Recall, while Tomek Links achieved marginally higher Precision. Despite these differences, the average performance, as measured by the F1-score, was comparable across the three methods. Notably, all three techniques resulted in approximately a 15% improvement in F1-score compared to the original dataset (FBER-o).

Swana E F, Doorsamy W, Bokoro P. Tomek link and SMOTE approaches for machine fault classification with an imbalanced dataset[J]. Sensors, 2022, 22(9): 3246.

-------------------------

[Comment #4] Line no. 362–399: Authors need to clarify whether the model’s precision and recall for blowing snow events (Class 1) were significantly affected by temporal data dependencies, considering the 13-year dataset.

[Response #4] Thank you for your valuable feedback. As you correctly pointed out, considering the 13-year dataset, it is indeed necessary to evaluate how the model's performance varies across individual years to ensure a more rigorous approach in dataset selection. In the revised manuscript, we have included the precision, recall, and F1-score results for each year, along with a comparison against the performance derived from the 13-year dataset. The detailed analysis and visual representation of these results have been added, as shown below.

-------------------------

(line 478)

As shown in Fig. 4, the yearly variation of the model's performance metrics is illustrated, based on data from the FBER station. The metrics, including precision, recall, and F1-score, are presented for both Class 1 (a) and Class 0 (b). In these figures, the solid lines represent the performance metrics for models trained on individual yearly datasets (Per-Year Data), while the dashed lines correspond to the metrics obtained from the 13-year aggregated dataset (13-Year Data). Additionally, the red bars indicate the number of snowdrift samples (Class 1) available in each year.

Fig 4. Yearly variation of model performance metrics based on FBER station data: (a) Class 1, (b) Class 0

From Fig. 4(a), it can be observed that the model's performance for Class 1 exhibits fluctuations in F1-score and recall across different years, which are closely related to the yearly variations in snowdrift sample size. For example, peaks in sample size, such as those in 2012 and 2017, correlate with higher F1-scores, whereas years with lower sample sizes, such as 2020, show a decline in these metrics. In contrast, Fig. 4(b) shows that the model's performance for Class 0 remains relatively stable across all years, with minimal variations in precision, recall, and F1-score. The consistency of these metrics indicates that the model's predictions for non-blowing snow events are less sensitive to temporal dependencies and sample size variability. Furthermore, as the sample size has a significant impact on the results, the 13-year dataset was selected as the training data, rather than using data from a single year or a subset of the years.

-------------------------

[Comment #5] Can the authors elaborate on how the non-linear impact of WS-MAX at low wind speeds (below 5 m/s) was addressed? Does this anomaly significantly affect model predictions?

[Response #5] Thank you for raising the issue regarding the non-linear impact of WS-MAX at low wind speeds (below 5 m/s) and its potential effect on model predictions. We provide further explanations on this below.

Point 1: (On the non-linear impact of WS-MAX below 5 m/s)

The observed non-linearity at wind speeds below 5 m/s primarily stems from the characteristics of the instrumentation. Upon carefully examining the training data, we identified a notable number of samples where snow flux was recorded as greater than zero, yet the corresponding maximum wind speed (WS-MAX) and mean wind speed output from the anemometer were still reported as 0 m/s. This anomaly can be attributed to two potential reasons:

- Instrument misreporting (false positives): As discussed in the manuscript, the Flowcapt instrument may occasionally misclassify snowfall or minor air disturbances as blowing snow events under zero wind speed conditions.
- Undetected low wind speeds: In some cases, actual blowing snow events might have occurred at very low wind speeds that were below the detection threshold of the anemometer, resulting in a recorded value of 0 m/s.

Both scenarios contribute to the presence of blowing snow event samples associated with 0 m/s wind speeds in the training data, which subsequently leads to the observed non-linear trend at wind speeds below 5 m/s.

Point 2: (On the impact of this anomaly on model predictions)

The feature interpretation provided in this study aims to objectively present patterns in the data. Removing these anomalous samples could risk introducing distortion into the dataset. Additionally, an examination of the confusion matrix reveals that the model's overall performance remains satisfactory despite the presence of this anomaly. It is important to note that this non-linear trend affects only a small segment of a single feature (WS-MAX) while the final predictions of the model are determined by the combined influence of all features.

We have incorporated a detailed explanation of the reasons behind the non-linear trend and its implications in the revised manuscript to address potential confusion.

-------------------------

(line 594)

Through an analysis of the mean curve in the one-way PDP presented in Fig 7(a), it is evident that when WS-MAX exceeds 5 m/s, there is a linear relationship between WS-MAX and the Partial Dependence, indicating that as the maximum hourly wind speed at the site increases, the probability of windblown snow occurrence proportionally rises. ~~Although an abnormal upward trend is observed in the low wind speed range (with WS-MAX approaching 0 m/s) in Fig 7(a), this effect can be considered negligible. The reason is that the new snowfall particles were counted as snow drifting because the FlowCapt sensor can detect snowflakes before a glycol/ethanol gauge records liquid equivalents. In reality, wind-blown snow primarily occurs at higher wind speeds, and the snow flux detected under low wind speed conditions does not represent actual windblown snow events. Therefore, the anomalous trend in the low wind speed range, caused by the high sensitivity of the instrument, can be disregarded.~~ However, an anomalous upward trend is observed in the low wind speed range (with WS-MAX approaching 0 m/s) in Fig. 7(a). This non-linearity can primarily be attributed to the characteristics of the instrumentation. Specifically, the FlowCapt sensor may misclassify snowfall or minor air disturbances as windblown snow events under near-zero wind speed conditions (false positives). Additionally, in certain instances, actual windblown snow events might occur at very low wind speeds that fall below the detection threshold of the anemometer, leading to samples where snow flux is detected despite a recorded WS-MAX of 0 m/s.

Despite this anomaly, its impact on model predictions is minimal. The low wind speed range constitutes only a small portion of the overall dataset, and the final predictions are determined by the combined influence of all features rather than a single segment of one feature. Furthermore, the confusion matrix confirms that the model performs well overall, suggesting that this localized non-linear effect does not significantly impair prediction accuracy. In reality, windblown snow primarily occurs at higher wind speeds, and the snow flux detected under low wind speed conditions does not reflect actual windblown snow events. Therefore, while the anomalous trend arises due to the high sensitivity of the instrument, it has negligible influence on the model's reliability and predictive capability.

-------------------------

[Comment #6] Line 483–537: While discussing the relationship between WS-AVG and blowing snow, could the diminishing impact beyond 10 m/s be attributed to hardware limitations of the FlowCapt sensor?

[Response #6] Thank you for raising the question regarding whether the diminishing impact of WS-AVG on blowing snow beyond 10 m/s could be attributed to hardware limitations of the FlowCapt sensor. Based on our analysis, we do not believe this phenomenon is due to sensor limitations. Instead, the diminishing impact is better explained by the physical relationship between wind speed and the conditions necessary for blowing snow events.

When the average wind speed reaches approximately 10 m/s, it already exceeds the threshold required to initiate and sustain blowing snow under most conditions. At this point, the probability of blowing snow occurrence has been substantially elevated, and further increases in wind speed do not lead to the same proportional rise in probability as seen at lower wind speeds. This reflects the saturation of favorable conditions for blowing snow, where the contribution of additional wind speed becomes less significant.

In response to your comment, we have expanded our discussion in the revised manuscript to provide a more detailed explanation of this trend.

-------------------------

(line 621)

In Fig 7(b), it is observed that similar to WS-MAX, an increase in WS-AVG also leads to a rise in the probability of wind-blown snow events. ~~However, it is also noted that when WS-AVG exceeds 10 m/s, the growth rate of the Partial Dependence significantly slows down. This suggests that while an increase in average wind speed within a certain range has a considerable impact on predicting windblown snow events, its influence diminishes as wind speed reaches a certain threshold. This phenomenon can be explained by the fact that, once WS-AVG surpasses a critical wind speed, other factors (such as air humidity and temperature) begin to significantly affect the formation and persistence of windblown snow. Therefore, although further increases in WS-AVG still promote the occurrence of windblown snow, its contribution gradually decreases, and the impact of other environmental factors becomes more pronounced.~~ Nevertheless, when WS-AVG exceeds approximately 10 m/s, the growth rate of the Partial Dependence curve slows considerably. This indicates that while increases in average wind speed play a significant role in predicting windblown snow events within a certain range, their influence diminishes once wind speed surpasses a critical threshold. This phenomenon can be attributed to the fact that, at wind speeds above 10 m/s, the conditions necessary to initiate and sustain blowing snow are already well met, and further increases in wind speed contribute less significantly to the probability of occurrence. At this stage, other environmental factors, such as air temperature and humidity, start to play a more prominent role in influencing the formation and persistence of windblown snow. Thus, although higher WS-AVG values still promote blowing snow events, their marginal contribution decreases as wind speed increases, highlighting the interplay of multiple factors in shaping these events.

-------------------------

[Comment #7] Line 558–588: In Fig. 8, the interaction of WS-MAX with AT and AH showed clear thresholds. How sensitive are these thresholds to the inclusion of additional meteorological features not considered in this study, like snow density or layer temperature?

[Response #7] The thresholds identified in Fig. 8, such as the significant increase in the probability of blowing snow when air temperature drops below -2°C or air humidity exceeds 70%, are likely tied to underlying physical phenomena and represent meaningful conditions for the occurrence of blowing snow. These thresholds provide valuable insights into the key meteorological conditions that promote blowing snow events.

However, it is challenging to evaluate how sensitive these thresholds are to additional features, such as snow density or layer temperature, due to the absence of these variables in the training dataset used for this study. That said, to our knowledge, the influence of such factors likely exists. Allow us to share preliminary findings from our recent, unpublished field research, where we investigated the role of snow density on blowing snow dynamics. Our results indicate that accumulated snow samples can be categorized into three distinct groups (Group A, B, and C) based on their snow density, with corresponding snow particle microstructure images highlighting these differences. Importantly, we observed that the threshold wind speeds required to initiate blowing snow increased progressively from Group A to Group C as snow density increased.

Moreover, in our ongoing research, we have found that interactions between different meteorological parameters can significantly influence blowing snow dynamics. For instance, higher air temperatures were observed to contribute to increased snow density, further altering the conditions for snow entrainment. These findings suggest that the interplay between various meteorological features warrants further exploration to comprehensively understand their collective impact on blowing snow events.

To address this limitation, we have expanded the Discussion and Conclusion section in the revised manuscript to acknowledge the absence of additional meteorological features, such as snow density and layer temperature, in our current analysis. We have also provided recommendations for future studies to incorporate these factors and investigate their potential influence on the thresholds identified in this study.

-------------------------

(line 753)

This study has achieved efficient prediction of snow drift events and exploration of feature impacts through machine learning methods, providing new insights and tools for research on snow drift disasters. However, limitations exist, primarily due to data constraints; for instance, features such as topography and snow layer temperature were not included in the analysis, which may affect the comprehensiveness of the results. ~~Future research directions could focus on data quality control to ensure the acquisition of more accurate meteorological data, enhancement of model accuracy by further optimizing existing models to accommodate more complex environmental conditions, and data enrichment by collecting additional variables and data under various meteorological conditions to improve the model's generalization capabilities. These measures will contribute to a deeper understanding and enhanced predictive capability for snow drift events.~~

Moreover, while the proposed prediction model demonstrates strong predictive capabilities within the range of historical meteorological data, its applicability to future scenarios, particularly under the influence of climate change, warrants further discussion. Climate change may lead to substantial shifts in key factors such as temperature, precipitation patterns, and wind dynamics, which could alter the occurrence and characteristics of snow drift events. These changes may reduce the reliability of the model if it is applied without adaptation to evolving environmental conditions. To ensure its continued applicability, periodic updates using newly collected datasets that reflect ongoing climatic trends are essential. Additionally, validating the model with contemporary observations and incorporating predictive climate models could enhance its robustness and adaptability to future scenarios.

Future research should prioritize data quality control to improve the precision of meteorological measurements, as well as expand the scope of analysis by including additional variables such as topography, snow density, and snow layer temperature. Enhancing model accuracy through the optimization of algorithms to account for more complex environmental interactions is another critical direction.

-------------------------

[Comment #8] Line 633–642: Considering the regional focus on the Alps, how generalizable are the model results to other geographies, such as polar regions with distinct meteorological patterns?

[Response #8] Thank you for raising this important question regarding the generalizability of our model results to other geographies, such as polar regions with distinct meteorological patterns. As our model is data-driven and grounded in objective meteorological observations, we believe it is conceptually applicable to other regions where blowing snow occurs. However, we acknowledge that differences in regional meteorological patterns, such as temperature extremes, wind dynamics, and snow properties, may affect the model's accuracy when applied beyond the specific conditions of the Alps.

To address this, future research should focus on validating the model in diverse geographical contexts, particularly in polar regions, by incorporating region-specific datasets and conducting comparative analyses. These efforts would help evaluate and, if necessary, adapt the model to account for local meteorological characteristics. In the revised manuscript, we have included a discussion of these limitations and highlighted the importance of extending the model's applicability through such targeted studies.

-------------------------

(line 773)

Future research should prioritize data quality control to improve the precision of meteorological measurements, as well as expand the scope of analysis by including additional variables such as topography, snow density, and snow layer temperature. Enhancing model accuracy through the optimization of algorithms to account for more complex environmental interactions is another critical direction. Finally, data enrichment by incorporating diverse datasets from various meteorological conditions will improve the model’s generalization capabilities. These efforts will ensure that the model remains relevant and reliable, providing an improved predictive capability for snow drift events.

-------------------------

[Comment #9] Improve the Figure no. 5 and 6 image quality

[Response #9] Thank you for your feedback. Both you and Reviewer #1 raised concerns regarding the clarity of Figures 5 and 6. We believe the issue arises from the lower resolution of the images in the initial submission, which were automatically generated by the Editorial Manager® system in the *PONE-D-24-48529.pdf* file. These images are preview versions with reduced clarity. However, if the figures are accessed directly by clicking the "*Click here to access/download; Figure; Fig 5.png*" link in the corresponding page, the original high-resolution source files of Figures 5 and 6 can be viewed, which exhibit excellent clarity.

Therefore, we anticipate that the issue of numbers being unclear in Figures 5 and 6 will not persist in the later stages of the review process. We have attached the high-resolution versions of Figures 5 and 6 below for reference in this resubmission.

If this does not address the concerns you have, we are more than willing to further revise Figures 5 and 6 based on any specific suggestions you may have.

-------------------------

(line 528)

Fig 5. SHAP Summary Plot：(a) FBER; (b) FGIE; (c) FHUE

Fig 6. SHAP force plot showing feature impacts for individual samples: (a), (b), (c) no-blown snow event, and (d) wind-blown snow event

-------------------------

[Comment #10] Authors may include a few more recent literatures on machine learning methods and XGBoost to strengthen the literature sections. Some are given below:

• doi/10.2166/hydro.2024.119/105935

• https://doi.org/10.2166/hydro.2024.292

• https://link.springer.com/article/10.1007/s11269-024-03879-9

• https://doi.org/10.1016/j.envsoft.2024.105971

• https://doi.org/10.1016/j.jhydrol.2021.126382

[Response #10] In our initial submission, we mentioned "5-fold cross-validation" multiple times but did not provide a detailed explanation, which might have caused confusion for readers. In the revised manuscript, we have addressed this by adding a clear introduction to 5-fold cross-validation to enhance the readers' understanding. Specifically, we referred to the comprehensive explanation of K-fold cross-validation techniques and their significance provided in the work of Baranwal et al. (2024) in Section 3.2 of their report, which served as a valuable reference for our revision.

-------------------------

(line 348)

During the training of the XGBoost model, adjusting multiple parameters is crucial for maximizing model performance, particularly to prevent overfitting and model complexity. To further optimize performance, this study employed GridSearchCV for grid search combined with 5-fold cross-validation. This technique divides the dataset into five equal subsets, where each subset is used as a validation set while the remaining four are used for training in an iterative manner. By cycling through all subsets, the method ensures that every data point is tested exactly once, providing a comprehensive evaluation of model performance (Baranwal et al., 2024). This approach ensures robust evaluation by mitigating overfitting and providing reliable performance estimates on unseen data. The grid search specifically targeted optimizing the following parameters: n_estimators (number of trees), max_depth (depth of trees), and learning_rate.

Baranwal A, Gaurav P, Reddy L, et al. Predicting temporal clear water scour depth around bridge piers with XGBoost and SVM–PSO approaches[J]. Journal of Hydroinformatics, 2024, 26(12): 3109-3129.

-------------------------

# Academic Editor

[Comment #1] Please ensure that your manuscript meets PLOS ONE's style requirements, including those for file naming. The PLOS ONE style templates can be found at

https://journals.plos.org/plosone/s/file?id=wjVg/PLOSOne_formatting_sample_main_body.pdf and

https://journals.plos.org/plosone/s/file?id=ba62/PLOSOne_formatting_sample_title_authors_affiliations.pdf

[Response #1] We have carefully reviewed and ensured that the current submission adheres strictly to PLOS ONE's style requirements, including the guidelines for file naming.

[Comment #2] PLOS requires an ORCID iD for the corresponding author in Editorial Manager on papers submitted after December 6th, 2016. Please ensure that you have an ORCID iD and that it is validated in Editorial Manager. To do this, go to ‘Update my Information’ (in the upper left-hand corner of the main menu), and click on the Fetch/Validate link next to the ORCID field. This will take you to the ORCID site and allow you to create a new iD or authenticate a pre-existing iD in Editorial Manager.

[Response #2] Thank you for the reminder. The corresponding author's ORCID iD has been successfully linked, and we are currently coordinating with the co-authors to complete their ORCID integration as well.

[Comment #3] Please note that PLOS ONE has specific guidelines on code sharing for submissions in which author-generated code underpins the findings in the manuscript. In these cases, we expect all author-generated code to be made available without restrictions upon publication of the work. Please review our guidelines at https://journals.plos.org/plosone/s/materials-and-software-sharing#loc-sharing-code and ensure that your code is shared in a way that follows best practice and facilitates reproducibility and reuse.

[Response #3] Thank you for highlighting the PLOS ONE guidelines on code sharing. We fully recognize the importance of providing author-generated code to ensure transparency, reproducibility, and reuse in research. In compliance with these guidelines, we have made the code used in this study available in the Supporting Information as S4 File: XGBoost-SHAP model implementation for blown snow detection.

-------------------------

(line 781 of the Revised Manuscript with Track Changes)

Acknowledgments

We would like to extend our sincere gratitude to the scientists and engineers involved in ensuring the continuous operation of measurements from the ISAW stations. The observational data collected from these stations can be accessed through the ISAW online database at <http://iav-portal.com/index.php?nav=iodmisawlist>. Additionally, the cleaned dataset in S Files 1, 2, and 3 and all author-generated code in S4 File are provided in the Supporting Information to support reproducibility and further research.

-------------------------

[Comment #4] We note that Figure 1 in your submission contain [map/satellite] images which may be copyrighted. All PLOS content is published under the Creative Commons Attribution License (CC BY 4.0), which means that the manuscript, images, and Supporting Information files will be freely available online, and any third party is permitted to access, download, copy, distribute, and use these materials in any way, even commercially, with proper attribution. For these reasons, we cannot publish previously copyrighted maps or satellite images created using proprietary data, such as Google software (Google Maps, Street View, and Earth). For more information, see our copyright guidelines: http://journals.plos.org/plosone/s/licenses-and-copyright.

[Response #4] Thank you for your suggestion. We have revised the base map in Figure 1. The updated base map is now sourced from OpenStreetMap, and the included map of Europe is obtained from the U.S. Geological Survey (USGS), following the recommendations provided by PLOS ONE at https://journals.plos.org/plosone/s/licenses-and-copyright. We hope that these revisions align with PLOS ONE's standards for content published under the Creative Commons Attribution License (CC BY 4.0).

Fig 1. Study area and locations of selected meteorological stations
